# Effectiveness of a physical exercise intervention program in improving functional mobility in older adults after hip fracture in later stage rehabilitation: protocol of a randomized clinical trial (REATIVE Study)

**DOI:** 10.1186/s12877-016-0370-7

**Published:** 2016-11-29

**Authors:** Camila Astolphi Lima, Catherine Sherrington, Adriana Guaraldo, Suzana Albuquerque de Moraes, Renata dos Ramos Varanda, Juliana de Araújo Melo, Kodi Edson Kojima, Monica Perracini

**Affiliations:** 1 Master’s and Doctoral Programs in Physical Therapy, Universidade Cidade de São Paulo, Rua Cesáreo Galeno 448, Tatuapé, São Paulo CEP: 03071-000 Brazil; 2 The George Institute for Global Health, Sydney Medical School, University of Sydney, Sydney, Australia; 3 Orthopedics and Traumatology Institute, Universidade de São Paulo, São Paulo, Brazil; 4 Master’s and Doctoral Programs in Gerontology, Faculty of Medicine, Universidade Estadual de Campinas, São Paulo, Brazil

**Keywords:** Hip fracture, Accidental falls, Rehabilitation, Exercise program, Clinical trial, Aging health

## Abstract

**Background:**

Hip fractures resulting from falls increase substantially with advancing age and less than half of older hip fracture survivors regain their former levels of mobility. There is increasing evidence that rehabilitation interventions with exercises that goes beyond the sub-acute phase or even in a later stage of care have a positive impact on various functional abilities. The purpose of this study is to determine if exercise program training for people who have suffered a fall-related hip fracture will improve functional mobility when compared with usual care.

**Methods:**

A randomized controlled trial with blinded assessors and intention-to-treat analysis will be undertaken. We will recruit 82 older adults, 60 years or over who have suffered a hip fracture due to a fall in the previous 6 to 24 months. Participants randomized to the Intervention Group (IG) will undertake a physical exercise program involving progressive and challenging balance training and neuromuscular and functional training of the lower limbs, conducted at home by physiotherapists, once a week, lasting about one hour, in the first, second and third month after randomization and will be taught to perform exercises at home, twice a week, using a booklet. Visits to monitor and progress the home exercise program will be conducted once a month, from the fourth to the sixth month and each 2 months until the end of the follow up at the 12^th^ month, i.e., a total of 18 sessions. Participants will receive monthly phone calls to encourage exercise adherence. The control group will receive usual care. The primary outcome will be mobility-related disability and participants will be assessed at baseline, and at 3 months, 6 and 12 months. Participants will receive monthly phone calls to ask about falls and exercise adherence. Adverse effects will be monitored.

**Discussion:**

This study proposes a home-based exercise program, which may in part overcome some barriers for rehabilitation, such as difficulties with public transportation and lack of a caregiver to accompany older patients to sessions. If a positive effect is observed this program has the potential to be incorporated into the public health system and contribute to building a pathway of care for older people with hip fracture.

**Trial registration:**

Clinicaltrials.gov Identifier: NCT02295527.

## Background

Over a lifetime, about half of women and a quarter of men will suffer a fragility bone fracture [1, 2], mostly due to falling [2]. Among the most serious and common fractures are hip fractures, which have significant risks of mortality [2] and disability [3]. It is estimated that in 2050 the global number of hip fractures will be between 7.3 and 21.3 million [4] and the cost with the treatment will be approximately of 131.5 billion US dollars [5].

Physical and psychological post-fracture limitations, such as decreased mobility [6] and lower limb strength [7], impaired balance [6], lack of confidence [7], fear of falling [6] and increased risk of falls [8] restrain about 40% of older people from returning to daily activities required to live independently and safely [3, 9].

Two years after injury more than half of older hip fracture survivors do not regain pre-injury functional abilities [10] and experience a moderate to high risk of further falls [6]. The restoration of mobility is crucial to enable old people to move around in and outside home, the recommended levels of physical activity, maintain social engagement and preserve autonomy. Ultimately, adequate mobility is a key component for an active and healthy ageing [11].

Although there is no consensus about the best intervention for improving functionality of older people after hip fracture [8], exercise programs delivered in an intensive and supervised basis, have been found to improve the mobility of these patients [8, 12, 13]. However, few studies have explored the late period of recovery (over 6 months) [14, 15], when functional limitations can persist due to inadequate dose as well as the type of exercise performed. Recently a home-based exercise intervention with minimal contact from a physiotherapist (4 visits) boosted by monthly telephone calls and the provision of a DVD version resulted in clinical meaningful improvement in the physical function of the lower limbs, but not in self-reported mobility and daily activities [16].

Over the next decades a huge increase in the older population will be experienced by low-and middle-income countries (LMICs), such as Brazil where population is ageing at one of the fastest rates in the world. The number of seniors in Brazil is predicted to double between 2002 and 2020, from 15 million to 32 million and to reach 64 million (30% of the total population) by 2050 [17]. Furthermore, people aged 80 and over will increase exponentially the next four decades, a growing and disproportionate demand on the health system and social support [17]. Along with an increased aged population LMICs will have to overcome barriers of care, such as difficulties in access and failure of existing health services to address older peoples’ rehabilitation needs. Yet post-fracture rehabilitation strategies are yet to be tested in low-and middle-income countries.

The primary aim of this study is evaluate the effectiveness of a physical exercise program for improving functional mobility in older people who have suffered a hip fracture due to a fall in the previous 6 to 48 months. Secondary aims to 1) determine the effects of the program in improving physiological risk for falls, functional and performance disability and quality of life; 2) establish predictors of adherence to the program and outcomes related to functional and mobility disability in older people after fall-related hip fracture.

## Methods

### Trial design

This is a randomized controlled trial with 12 months follow-up. The Ethics Committee on Human Research of the City University of São Paulo (CAAE: 27398814.7.0000.0064) approved the project. This study has been registered with the number protocol of NTC02295527 trials register.

After the baseline assessment an independent investigator, not involved with recruitment or data collection, will randomly allocate the eligible participants to one of two parallel groups by opening a sealed envelope with study group assignments i.e., a concealed allocation system. The randomization schedule will be prepared in advance by an independent investigator, not involved with recruitment or data collection, using in randomly-ordered blocks of 2, 4, and 6 participants. All assessors will be blinded to the group allocation, however, due to the trial design, participants and therapist are not able to be blinded to group allocation.

### Participants

Community-dwelling people aged 60 years and over, men and women, after a first hip fracture due to falling or bone fragility that has been surgically treated will be recruited. These participants will be in the later stage of rehabilitation phase (6 months to 2 years after the fracture). Participants will be recruited from public and private hospitals in São Paulo municipality, from rehabilitation services and through general advertisement.

Participants will be excluded if they have a severe cognitive impairment (assessed using the Mini Mental State Examination, with scores adjusted according to educational level [18] illiterate ≤ 13 points, 1 to 8 years of schooling ≤18 points and, 9 or more years of schooling ≤26 points); inability to walk despite using a walking aid; progressive or severe neurological disease (e.g., Parkinson’s disease, stroke) with severe functional limitations; communication disability that would impede intervention participation (severe uncompensated visual or hearing deficits); medical condition contraindicating exercise (e.g. unstable angina, severe heart disease, large or expanding aortic aneurysm, etc.); acute vertigo or dizziness less than 3 months duration; engaged in a regular exercise program with a frequency of at least twice a week 30min a day (excluding walking and senior dance).

### Interventions

#### Intervention group

Intervention Group (IG) will undergo a physical exercise program involving progressive and challenging balance training and neuromuscular and functional training of the lower limbs, conducted at home by physiotherapists, once a week, lasting about one hour, in the first, second and third month after randomization and will be taught to perform exercises at home, twice a week, using a booklet.

Visits to monitor and progress the home exercise program will be conducted once a month, from the fourth to the sixth month and each 2 months until the end of the follow up at the 12^th^ month, i.e., a total of 18 sessions. Participants will receive monthly phone calls to encourage exercise adherence. The physical exercise program will consist of balance training and neuromuscular training of lower limbs, as described below:

- *Progressive strengthening of three muscle groups: ankle dorsiflexors, knee extensors and hip abductors.* The intensity will be defined individually and will correspond to the adequate amount of weight that would allow them to perform for 10–15 reps, 2–3 sets. Participants will receive ankle cuff weights to perform the exercises.
- *Progressive balance training*, consisting of a protocol of exercises with 1 or 2 sets of 10 repetitions, according to individual capacity, to ensure that participants reach their maximum level of functionality [19]. Weight-bearing exercises [14, 15], training with emphasis on perception of the limits of stability, change of position/direction, maintenance of static and dynamic stability, anticipatory adjustments using different bases of support and different sensory conditions will be made. Exercises will include 360° turns, sitting and standing, walking up and down stairs, functional reach, steps in different directions and walk training.

In the first session the physiotherapist will choose two exercises that best address the participant’s functional goals and will add more exercises each week according to the participant’s ability, rate of improvement and motivation. The degree of balance challenge will be progressed in terms of reducing the base of support, the use of hands and the inclusion of multisegmental coordination exercises. Participants will be receive recommendations on how to perform the exercises safely, such as standing near of a table or in the corner of a room, so they can promptly use hands for protection if they start to fall. Those who are frailer will be encouraged to perform exercises under supervision of a caregiver. Participants will receive a detailed booklet, containing photos and instructions for the exercises.

Adverse effects related to the intervention that interfere in the activities of everyday life for more than 48h or require medical attention will be recorded and monitored (pain, discomfort, swelling, etc.). During home-visits the physiotherapist will check how the participant is performing the exercises and progress the exercises if necessary. Participants will receive monthly phone calls to encourage exercise adherence and to collect information about falls and their consequences. Participants will be asked reasons for non-adherence to exercises.

Participants will receive a diary logbook to record the number of falls a week and a guidance booklet containing information about prevention of falls (involving person, environmental and occupational factors), prevention of fractures and bone health. This group will also receive usual health care.

#### Control group

This group will receive usual care; so will not be disadvantaged by participating in the study. Usual care will be what the participant receives as part of the network of health care which he/she is part of. The participants will receive a diary falls logbook and a guidance booklet containing information about prevention of falls, fractures and bone health. Control group participants will receive monthly phone calls requesting information about falls and their consequences.

### Outcomes

All participants will be assessed in their homes at four different timepoints: at baseline and after 3, 6 and 12 months of randomization.

#### Primary outcomes

Lower limb functionality will be measured by the Short Physical Performance Battery (SPPB) [20], that consists of three tests that assess static balance, walking speed and, indirectly, the strength of the lower limbs (sitting and standing from a chair unassisted). Each test has a score of zero (worst performance) to 4 points (best performance), totaling a maximum final score of 12 points. This instrument can predict negative consequences such as falls and the onset of disability in older people [21]. The use of walking aids will be allowed during the test if necessary.

#### Secondary outcomes

*Physiological risk of falls* will be measured by the Profile Physiological Assessment long version (PPA) [22, 23] which provides an overall score for risk of falling through a series of tests that evaluates the systems involved in the postural stability system. The results are computed using software specifically developed for the test that generates a report assessing the risk of falls. Physiological PPA tests include:

- ○ Vision: low and high contrast, visual acuity and contrast sensitivity evaluated by the Melbourne Edge Test;
- ○ The peripheral sensation tests: tactile sensitivity, measured by Semmes-Weinstein-type pressure esthesiometer;
- ○ Proprioception: position sense of lower limbs;
- ○ Muscle strength test: the muscle groups flexor and extensors of knee and ankle dorsi flexors will be assessed through a dynamometer;
- ○ Reaction time test: Reaction time is measured in milliseconds using a modified computer, a mouse is used for response to finger pressure task and one pedal pressure for feet task;
- ○ Balance test through a portable stabilogram, the swaymeter.

*Functional performance* measured by the self-reported WHO Disability Assessment Schedule (WHODAS 2.0) [24]. This scale measures health and functionality in 6 domains: cognition, mobility, self-care, interpersonal relationships, daily activities and participation. It was developed by WHO based on the International Classification of Functioning, Disability and Health (ICF), and it is not intended for any specific health condition. This short version assesses the difficulty degree of performing 12 activities in past 30 days, which can be classified as none, mild, moderate, severe and extreme (cannot be performed). The degree of difficulty reported is based on the presence of increased effort, discomfort or pain, slowness or the presence of some change in the way of doing the activity.

*Quality of life* measured by WHOQOL-bref [25], consists of 26 questions divided in 4 domains: physical, psychological, social relationships and environment. The questionnaire is based on the last 2 weeks of the participant and evaluated, for example, how safe he/she feel in her/his daily life and how satisfied is he/she with his/her ability to perform daily life activities.

*Physical activity level* will be measured by the self-reported Incidental and Planned Exercise Questionnaire - IPEQ_W [26], ten questions designed to measure physical activity planned and unplanned for older people. The version estimates the intensity of physical activity during the last week will be used. The participant will be questioned for the frequency (every day, 3–6 times per week, twice a week, once a week or less than once per week) and duration of activity (less than 15min per day, more than 15min and under 30min per day, more than 30min and less than 1h, less over 1h and 2h per day more and less than 4h per day 2h, 4h, and daily or more). The total score is derived by multiplying the frequency categories by categories of activity duration, expressed in hours per week.

*Occurrence of falls* will be assessed by monthly phone calls to participants who will be encouraged to use to diary to record the number of falls each day. A structured questionnaire will be used to identify possible falls and their consequences. The intervention group will be compared to control group at 6 and 12 months follow-up in relation to the number of falls (primary outcome) and the proportion of fallers.

#### Sample characterization

*Functional Comorbidity Index* [27] is an 18-item scale designed to assess comorbidities related to physical function rather than mortality. Each scale item receives 1 point if the patient reports the disease.

*Cognitive function* will be assessed through the Montreal Cognitive Assessment Test (MoCA Test) [28], a battery of tests to detect mild cognitive impairment. The test takes 10min and the maximum score is 30 points. The test assesses different cognitive domains: attention and concentration, memory, language, visuoconstructional skills, conceptual thinking, calculations and orientation.

*Depressive symptoms* assessed by the Geriatric Depression Scale (GDS) [29], an instrument designed to detect depression in the older people. The short version contains 15 items and is the most used in clinical settings due to its rapid application. Each answer has a score of 0 or 1, and a final score above 6 points is considered to be indicative of depression.

*Fall-related self-efficacy*, assessed by the Falls Efficacy Scale International (FES-I) [30] a scale of self-efficacy of fall avoidance during activities of everyday life, which has 16 items with scores ranging from 1 to 4 (maximum total score 64) and a cutoff score of 31 points.

*Nutritional status*, assessed by the Mini Nutritional Assessment (MNA) [31] a questionnaire consisting of two parts: Screening and Overall Assessment. The screening consists of 6 basic questions about weight loss, BMI, mobility and neuropsychological problems. The score in the first part of the assessment can vary from 0 to 14 points. If the score is less than 12, the researcher must continue through the second part of the questionnaire, composed of more specific questions on nutrition and physical assessment. The sum of 18 questions can provide a maximum score of 30. The person with a score greater than or equal to 24 points is considered well-nourished, a score of 17–23.5 is considered at risk for malnutrition, and a score of less than 17 classifies the person as malnourished.

*Number of regular medications*, prescribed by a doctor, in the last 3 months will be computed, except vitamins and other medications for sporadic use (analgesics, for example). The use of psychotropic medications will be specifically recorded.

*Hospitalization and care in emergency department*: duration and reason for hospitalization and duration of stay in the emergency department during the study period.

### Sample size calculation

The sample size of this study (41 participants per group) is sufficient to detect a change of 1.0 ± 1.48 points in the score of the Short Physical Performance Battery SPPB [32] with a statistical power of 80% and a significance level of 5%, considering a possible sample loss of up to 15%.

### Statistical analysis

The comparison between groups on tests involving continuous variables (lower limbs functionality, physiological risk of falls, functional performance, quality of life and physical activity level) will be performed using linear models (General Linear Models-ANCOVA) adjusted for baseline values. The rate of falls will be compared between groups using negative binomial regression. Logistic regression models will be used to compare dichotomous variables between groups. All analyses will use the principle of intention-to- treat, in that participants will be analyzed according to their randomized assignment, regardless of whether they complied with the study protocol.

## Discussion

Several studies have found exercise in the late phase of recovery after hip fracture to be effective in improving mobility, balance [14, 15] and muscle strength [7, 14, 15]. However no previous studies have been conducted in low- and middle-income countries (LMICs). This study aims to evaluate the effectiveness of a physical exercise program in a later stage of recovery after fall-related hip fracture in improving functional mobility in older people when compared with usual care. If a positive effect is observed, this study will contribute to increase the evidence about the functionality of the therapeutic exercise program to be tested, functional training focusing on the progressive balance and resistance training of lower limb.

In LMICs the impact of ageing population and the burden of non-communicable diseases and injuries will be very significant in coming years. The evaluation of the effectiveness of an exercise program in late rehabilitation after hip fracture is substantially important in health care settings in these countries where the provision of rehabilitation after hip fracture in acute and sub-acute phases is extremely variable and restricted. Although in Brazil the hip fracture has been chosen as a sentinel event for falls in the elderly [33], the establishment of a pathway of care after hip fracture is still nascent and fragmented, with negative consequences for efficiency and equity access to rehabilitation. There is a need to organize a continuum of structured networks through integrated care health care, ensuring the transition from hospital to home care and outpatient.

We are proposing a home-based exercise program, which may in part overcome some barriers for rehabilitation, such as difficulties with public transportation and lack of a caregiver to accompany older patients to sessions. Although the program are supported by supervision on a regular basis, we believe that the visits distribution along the follow up will help patients to cope with the exercises, particularly those who are illiterate or less motivated. If proven to be effective this program may be incorporated into the public health system and contribute to building a pathway of care for older people with hip fracture.
